# RISKY BEHAVIORS WITH NONALCOHOLIC FATTY LIVER (NAFLD) AND NONALCOHOLIC STEATOHEPATITIS (NASH) AMONG OLDER ADULTS

**DOI:** 10.1093/geroni/igad104.2520

**Published:** 2023-12-21

**Authors:** Tung-Sung Tseng, Wei-Ting Lin, Peng-sheng Ting, Chiung-Kuei Huang, Po-Hung Chen, Hui-Yi Lin

**Affiliations:** LSUHSC, New Orleans, Louisiana, United States; Tulane University, New Orleans, Louisiana, United States; Tulane University School of Medicine, New Orleans, Louisiana, United States; Tulane University School of Medicine, New Orleans, Louisiana, United States; Medicine, Johns Hopkins University, Baltimore, Maryland, United States; LSUHSC, New Orleans, Louisiana, United States

## Abstract

**Background:**

Nonalcoholic fatty liver disease (NAFLD) and nonalcoholic steatohepatitis (NASH) are emerging public health concerns among older adults, particularly those with risk behaviors.

**Objectives:**

We evaluate the association between risk behavior and the prevalence of NAFLD and NASH in older adults.

**Methods:**

A total of 1444 US individuals aged 60 years and older were included from the 2017- 2020 NHANES survey. Dietary, Sugar-sweetened beverages, smoking, physical activities, stiffness, controlled attenuation parameters, alanine aminotransferase (ALT), and aspartate aminotransferase (AST) were measured. We performed multivariable logistic regression models with interaction terms and stratified analysis to assess the interplay effect between soda intake and body mass index (BMI) on liver stiffness measurements. All statistical analyses were under survey modules with an appropriate sampling weight.

**Results:**

The prevalence of NAFLD/NASH varied significantly by age, gender, race, alcohol use, BMI, total energy consumption, ALT, and (all p≤0.001). The results showed that several risk behaviors, including smoking, physical inactivity, and sweetened beverage consumption, were not significantly associated with developing and worsening NAFLD and NASH among elderly individuals. However, higher total energy consumption, obesity, and alcohol use were risk factors for NAFLD/NASH.

**Conclusion:**

Our study findings highlight the importance of promoting some healthy lifestyle behaviors among elderly individuals to prevent the development and progression of NAFLD and NASH. Further research is needed to investigate optimal strategies for promoting healthy beh

